# Correction: Influence of carbonization conditions on luminescence and gene delivery properties of nitrogen-doped carbon dots

**DOI:** 10.1039/c9ra90019e

**Published:** 2019-03-07

**Authors:** Mickaël Claudel, Jiahui Fan, Mickaël Rapp, Françoise Pons, Luc Lebeau

**Affiliations:** Laboratoire de Conception et Application de Molécules Bioactives, UMR 7199 CNRS – Université de Strasbourg, Faculté de Pharmacie 74 Route du Rhin – BP 60024 67401 Illkirch France llebeau@unistra.fr

## Abstract

Correction for ‘Influence of carbonization conditions on luminescence and gene delivery properties of nitrogen-doped carbon dots’ by Mickaël Claudel *et al.*, *RSC Adv.*, 2019, **9**, 3493–3502.

The authors regret that their names were displayed incorrectly in the original article. The correct author names are as presented above.

The Royal Society of Chemistry apologises for these errors and any consequent inconvenience to authors and readers.

## Supplementary Material

